# Repeatable Imaging of Soil Processes Through a Stabilized Port: Examples of (i) Soil Contaminants and (ii) Plant Root Growth

**DOI:** 10.3390/s25030968

**Published:** 2025-02-06

**Authors:** Julio A. Zimbron, Christian C. Rayo

**Affiliations:** E-Flux, LLC, 3185A Rampart Road, Fort Collins, CO 80521, USA; crayo@soilgasflux.com

**Keywords:** soil imaging, NAPL, root growth, UV light

## Abstract

This work presents an imaging testing system (software and hardware) that can generate repeatable images through a stabilized port in the soil for processes known to change with time. The system includes (i) a stabilized port in the ground made of standard PVC pipe, with sections lined with a borosilicate glass tube, and (ii) a digital imaging instrument to survey the optically transparent portion of the stabilized port. The instrument uses a probe containing a digital camera and two light sources, one using white lights and one using ultraviolet (UV) lights (365 nm). The main instrument controls the probe using a cable within the stabilized port to take overlapping pictures of the soil under the different light sources. Two examples are provided, one to document the distribution of soil and groundwater contaminants known as non-aqueous phase liquids (NAPL, which include petroleum) at variable water saturation levels and a second one to monitor the growth of a plant over a 2-week interval. In both examples, the system successfully identified critical changes in soil processes and showed a resolution of approximately 15 µm (in the order of the thickness of a human hair), demonstrating the potential for repeated imaging of soil processes known to experience temporal changes. Both examples are illustrative, as additional applications might be possible. The novelty of this system lies in its ability to generate repeated measurements at larger depths than the current shallow systems installed by hand.

## 1. Introduction

The study of soil processes is limited by the complexities of soil geology and the lack of direct access to locations where these processes occur. Soil is often formed of layers of different materials deposited over different long-term geological times and experiences the flow of immiscible fluids (water, air, and non-aqueous phase liquids, known as NAPL, which include petroleum and other liquids). The complexity of the soil media, in addition to multiphase fluid transport in soils, the small soil pore scale, and the variable time scale over which these processes occur creates a challenging problem [1].

Much of the study of soil properties and soil processes is performed by extracting soil cores and analyzing them extensively for particle size distribution (soil type), water and contaminant content [2], computer tomography imaging [3], soil geochemistry/mineral content [4], and high-resolution imaging [5]. In addition to the disturbance of the soils during sampling (which results in the recovery of smaller cores than those of the length surveyed) [2], these properties, although extremely important, are only measured once, as the samples are destroyed after analysis (destructive testing).

Geophysical tests exist that offer field measurements indirectly related to bulk (average) soil properties (over a soil interval) [6]. These include electrical resistivity, electromagnetic measurements, ground-penetrating radar, and others. These methods have proven useful for specific applications and can be repeated at different times but have the disadvantages of not providing a direct measurement of specific soil properties (thus often requiring empirical correlations to estimate meaningful soil properties) and averaging those measurements over a resolution several orders of magnitude larger than that of the pore scale [6].

Imaging technologies have proven useful in the study of soil processes in the field. For example, technologies classified as high-resolution site characterization (HRSC) tools aid in the detailed characterization of soil and soil contamination using probe-mounted sensors that collect data as the probe penetrates the ground [7,8]. HRSC tools include laser-induced fluorescence (LIF) [7,8] and the optical image profiler (OIP) [9], which can help determine vertical profiles of the contaminants based on their response to UV lights. The HRSC approach, with its high data density (generally ~one data point every 1–2 inches), has led to better soil and contaminant characterization and more effective remedies to mitigate the contamination risks [10]. HRSC is recognized as a best management practice (BMP) for systematic contaminated site management [7]. Despite their proven advantages, HRSC technologies have limitations. Their use involves ground-penetrating probes, which create challenges such as (i) repeated use requiring heavy equipment redeployment; (ii) soil disturbance upon ground penetration and retraction, which affects measurement reproducibility; and (iii) high costs. Consequently, most HRSC surveys are conducted as one-time snapshots. NAPL distributions in soils change with time, influenced by factors like groundwater fluctuations, contaminant mass depletion, and changes in hydraulic head [10]. These dynamics can result in contaminants suddenly appearing in previously unaffected monitoring wells or migrating into adjacent properties [10]. Current HRSC tools can provide spatially dense datasets, but not temporally dense datasets.

An alternative approach for imaging soils has been used to study plant roots using clear glass tubes and small imaging devices called rhizotrons, which have been used for repeated surveys of the plant roots. Different rhizotron versions use small cameras and light sources, deployed within tubes with diameters between 50 and 100 mm, to image the soil and study plant roots within [11]. Practical limitations of this approach are the tube installation (suitable to depths~<1 m) and those inherent to the sensor (excitation wavelength of the light source, sensor resolution, image storage capacity, etc.).

In this manuscript, we present a novel approach that addresses the limitations of current soil monitoring approaches by providing a test that can be repeated on demand using a portable tool that can reach larger depths than those possible by installation by hand. The tool is based on digital imaging of the soil using a camera and a set of light sources that are deployed within a hollow pipe (a dry well casing) fitted with a section of clear borosilicate glass. The dry well casing is made of standard PVC material used for groundwater and monitoring well installations and can be deployed in the soil using standard direct push drill rigs and drilling tools, which can go down to depths of up to 50 ft or more. The glass piece is fitted within the PCV pipe using watertight connections. The PVC provides protection to the glass during handling and field installation. Cut-out sections in the PVC allow for digital imaging of the soil through the borosilicate tube.

This report describes the instrument and presents two early use examples for different applications: (i) the study of soil contaminant distributions and (ii) the study of plant root growth. Additional applications might be possible using multispectral analysis, for example, the detection of fungal infections in roots [12,13], the measurement of soil carbon content [14], and others ([11] and references therein). The novelty of this system lies in its ability to generate repeated measurements of processes suitable for imaging at larger depths than the current shallow systems installed by hand.

## 2. Materials and Methods

### 2.1. Overview

**Figure 1:**
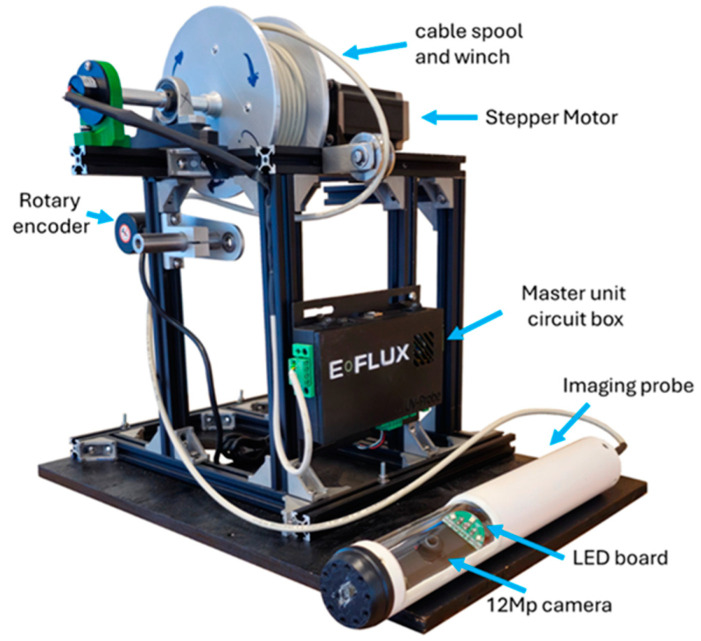
The field portable instrument is composed of a Main Unit and an Imaging Probe.

The instrument described in this work allows the collection of pictures below the ground surface using a proprietary design ([15], E-Flux, Fort Collins, CO, USA). The apparatus consists of an Imaging Probe that includes light sources and a camera that takes pictures of the soil. The light sources comprise LEDs (currently two sets, one of white lights and one of a 365 nm UV light, from CreeLED, Inc., Durham, NC, USA and Luminus, Inc., Sunnyvale, CA, USA, respectively) to enable imaging under different excitation wavelengths. Additionally, the system has a Master Unit and a Graphic User Interface (GUI), which coordinates the probe movement, the collection of images, and defines the vertical interval of the domain to be surveyed. Figure 1 shows a picture of the Imaging Probe and the Master Unit. The probe includes a 12 MP (megapixel) camera (Arducam Technology Co., Ltd., Hong Kong, China), which uses a SONY IMX477 sensor (Sony, Kumagoto, Japan). The camera is fitted with a 140° (fisheye) lens and an LED board. The probe is lowered through a cable into a dry well made of standard PCV casing with clear openings lined with borosilicate glass (see the following section for a complete description). A microcontroller (Arduino, Lombardia, Italy) controls the probe and a cable spool to lower the probe. The probe is aligned with the glass openings in the dry well by a magnetic field created by magnets mounted in the probe and on the dry well. A portable computer connected to the microcontroller runs a graphical user interface (GUI) to program the system and display the status of the Imaging Probe.

### 2.2. Field Hardware and Installation

**Figure 2:**
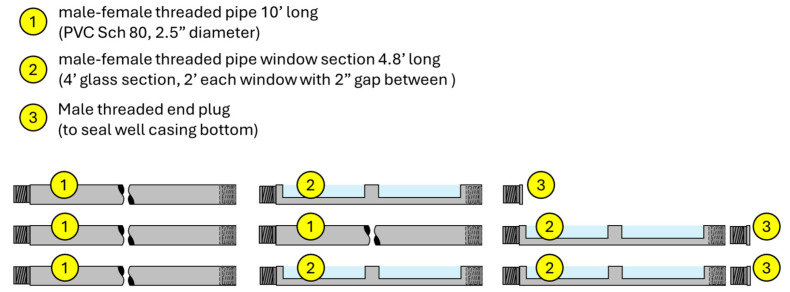
Well casing modular design and configuration examples. Note that the illustration shows materials lying horizontally, although field installations are vertical.

Figure 2 shows the well modular design and possible configurations, made of nominal 2.5″ Schedule 80 PVC pipe, using threaded connections. All threads are compatible with standard PVC pipe Sch. 80 used for well casing (IPEX, Oakville, ON, Canada). The main components of this design are as follows: (i) a 305 cm (10′) long male-female threaded pipe; (ii) a 146 cm (4.8′) borosilicate glass section (Pegasus Glass, Cambridge, ON, Canada), encased in a PVC pipe custom machined to create a seal around the glass (openings in the PVC pipe are 61 cm (2′) long and a 5.1 cm (2″) gap between both sides); and (iii) an end plug male threaded to seal the well casing. These pieces allow the pipe assembly to be installed at different depths depending on the required installation needs. Note that the surveyable depth is based on the 146 cm (4.8′) borosilicate glass, but multiple glass sections can be stacked. The design results in an assembly similar to those used for standard well installations in the field. Field installation requires a direct push rig, which provides a hole stabilized in the ground by the hollow drilling tool [16]. Once the drilling tool has reached the desired depth in the soil, the assembly is dropped inside, and the tool is retracted to allow the native soil formation to collapse around it. The disruption of the soil during installation is minimized by the selection of a drilling tool to minimize the gap between the inner diameter of the drilling tool and the well casing and does not occur afterwards during the actual surveys. This level of disruption might be similar to other soil surveyance methods, such as probe-mounted HRSC sensors [7], monitoring wells [6], rhizotrons [11], and extraction of soil cores for lab analysis [6]. The use of hollow stem auger (HAS), focused on preserving soil cores, might result in an even larger disturbance of the native soil (for example, see [2]). Note that the lab experiments described in this work did not require the heavy equipment described here for field installation.

The outer surface of the glass installed in this manner is in direct contact with the soil, leaving the inner surface clean and suitable for imaging. Condensation of water in this inner surface is avoided by having a dry well installation; in case the well casing were to leak, the water inside would need to be pumped out and condensation within cleaned with a cloth and solvent (methanol). This situation has been observed in some field applications of this system.

### 2.3. Instrumentation

#### 2.3.1. Graphical User Interface (GUI)

**Figure 3:**
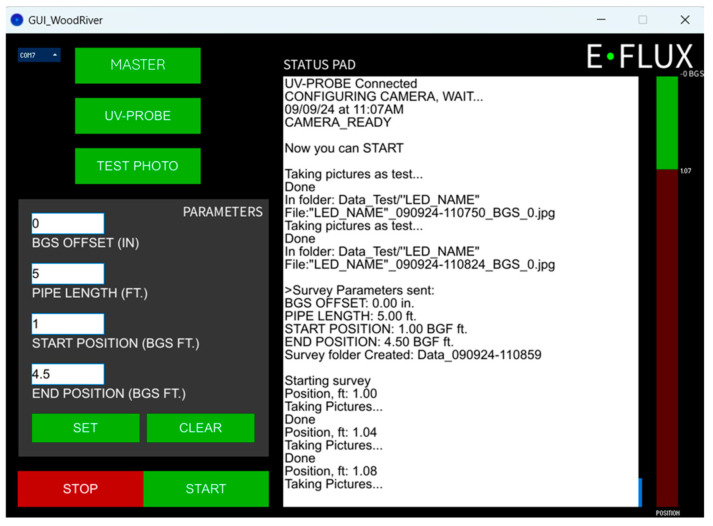
Screenshot of the Graphic User Interface (GUI).

The GUI runs on a laptop with Windows 10 OS. The GUI input parameters include the dry well vertical dimensions and the clear portion of the dry well to be surveyed. It is typical for well casing materials to stick above the ground surface and to code the position along the well casing using length units below the ground surface (BGS). The surveys use the ground surface as a datum, with the starting probe location at the open top edge of the well casing. The difference between the ground surface and the upper open edge of the well casing is entered as the offset (as a negative length). Hypothetically, a positive offset would indicate if the top of the well is below ground surface (for example, locations secured through a metal vault finished at ground surface, common for areas with active traffic). The GUI also reports the status of the current survey (the current probe location). A log file is saved on the laptop computer with all the parameters shown in the Status Pad of the GUI (Figure 3).

#### 2.3.2. Master Unit

**Figure 4:**
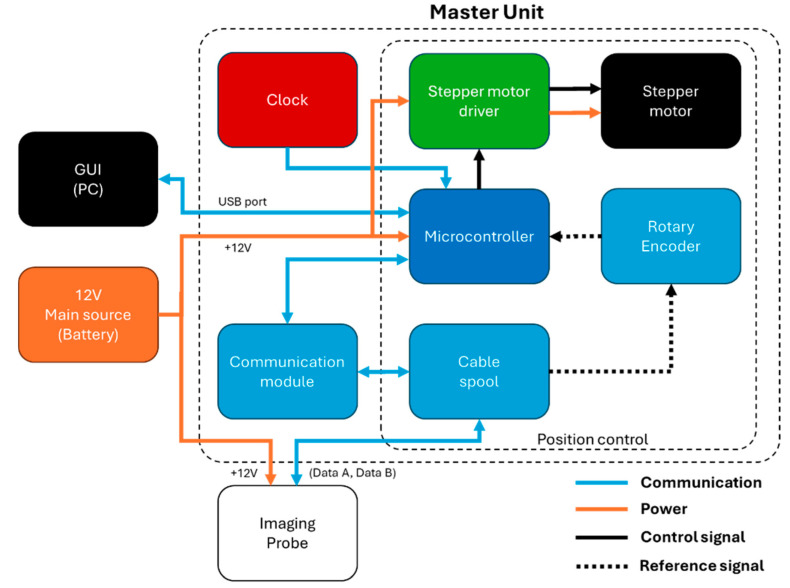
Master Unit block diagram and its relationship to the GUI (located on a portable computer) and the Imaging Probe.

The Master Unit manages the Imaging Probe to coordinate its movement and the imaging capture, with a major goal of achieving repeatable surveys. The main parts of the Master Unit are the Position Control and the Communications Module, as shown in Figure 4. The Position Control uses a proportional and derivative (PD) controller that receives feedback from the Rotary Encoder (Jinan Kesheng Automation Tecnology, Jinan, China) to measure the length of cable delivered by the cable spool. The Rotary Encoder is attached to a pulley. The pulses of the Rotary Encoder and the pulley dimensions are used to calculate the length of cable fed through the pulley and the position of the camera, which is then updated in the GUI display. The unit is programmed to move 12.7 mm (0.5″) at a time. The Rotary Encoder, using a 600 ppr (pulses per revolution) and attached to a 30 mm spool diameter, allows a length resolution of 0.16 mm per pulse read, equivalent to 81 pulses per step. The Imaging Probe takes two pictures at each position, one under white lights and then one under UV lights, before moving to the next position to repeat the cycle until the bottom of the surveyed interval is reached.

The Communication Module coordinates communications between the Master Unit and the Imaging Probe. It uses a transceiver MAX485, which runs a half-duplex transmission mode. The Master Unit commands the Imaging Probe to take each picture. The file name for each picture uses the date, time, depth, and light source used. The time stamp of the Imaging Probe is reset using the Master Unit clock time (since the probe time is lost each time it is turned off). The depth used for the file name is updated at each step using the value from the Master Unit.

#### 2.3.3. Imaging Probe Control

**Figure 5:**
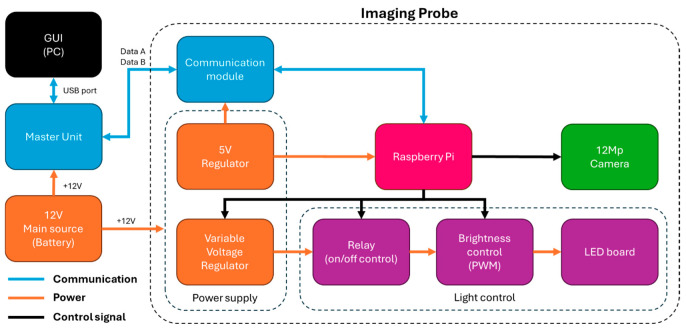
Imaging Probe block diagram.

The Imaging Probe includes the camera and light sources. The probe includes a local microprocessor (Raspberry Pi Zero 2 W, Cambridge, UK) and takes pictures triggered by the Master Unit. The control diagram is shown in Figure 5. It is programmed using Python 3 (version 3.11.9) to control the light sources and the operating parameters of the camera (specific to each light source). The Imaging Probe has two voltage regulators: (i) a 5 V regulator for the Raspberry Pi and the Communication Module, and (ii) a variable voltage regulator that provides the specific voltage required by the LEDs. The current design of the Imaging Probe includes two different light sources: (i) a white light (4000 K color temperature, or neutral white) and (ii) a UV (365 nm) light source. Each light source consists of two sets of LEDs, centered around the camera to minimize shadows and glare reflections. Each of the top and bottom halves of both light sources is mounted on a single LED board. The white light source has one LED on each LED board, while the UV light source has two LEDs on each LED board. The white LEDs require 3.05 V and draw 350 mA (for a total of 700 mA), and UV LEDs require 3.7 V and draw 500 mA (for a total of 2000 mA). A relay array helps select the light source to use for each picture. A controller regulates the light intensity using pulse width modulation (PWM), which is made through a MOSFET (DMT6009LCT) to control the duty cycle.

The 12 MP camera captures the picture, with custom settings for ISO (the sensitivity of the camera sensor) and exposure time, controlled using the Picamera2 library (version 0.3.16), which runs on Python 3 [17]. Custom settings were optimized for imaging using each of the two light sources and held constant throughout the entire survey for each type of light. These parameters are shown in Table 1. The camera used is placed at a focal distance of 17 mm from the soil, with an effective 98.5° field of view (FOV) capturing pictures of 49 mm width, covering approximately 27% of the whole glass tube circumference.

### 2.4. Power Consumption

The power Main Source uses a 12 V, 240 Wh (Watts-hour) portable battery (CTECHI, Shenzhen, China). The probe consumed a peak current of 0.2 A while surveying with white lights, while surveying under UV lights required 1 A. The Master Unit had a 1.3 A consumption rate (12 V). The minimum current for the entire system required 2.4 A. Over a full surveyable length of 1.46 m (4.8′), the power consumption is 53 Wh, taking 27.5 min. The surveying speed is 3.2 m/h (10.52′/h). For example, two clear sections (146 cm each) could be surveyed in ~1 h. A full battery charge would be sufficient to survey a total length of 14.3 m (47.4′, nearly 10 clear sections) in 4.5 h. The main features of the instrument are summarized in Table 2.

### 2.5. Post Processing Software

**Figure 6:**
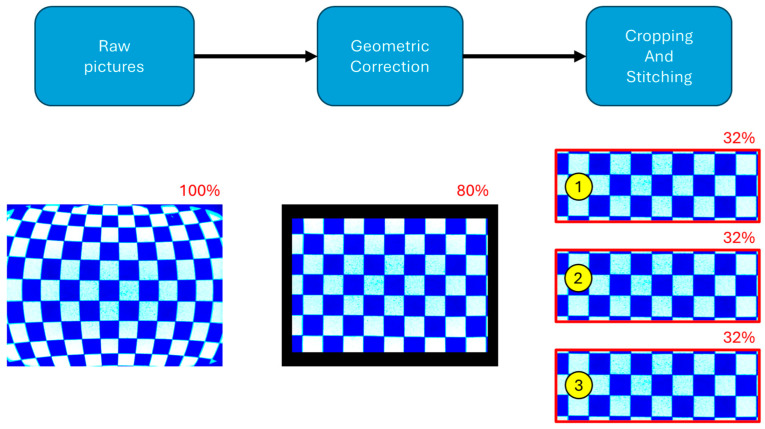
Post-processing block diagram and example using a checkerboard for calibration. The percentages shown indicate the image height preserved in each step, with respect to the original, raw image.

Images were saved locally to the probe for later post-processing. Real-time imaging is possible. Post-processing consists of a series of adjustments of the raw pictures taken by the camera to arrive at a curated form of data. This sequence is shown in Figure 6. Postprocessing is conducted on an external computer after extracting the picture files from the Imaging Probe. Image processing is conducted using Python 3 (version 3.11.9) and the Open-cv library (version 4.8.1). An initial correction recovers the original, rectangular geometry around the glass cylinder circumference, and a cropping/stitching obtains the full image of the viewable section of the dry well installation.

#### 2.5.1. Geometric Correction

The digital images collected by the probe suffer from a geometric deformation due to (i) the geometry of the curved glass well and (ii) the wide-angle (140°) lens used. This deformation was corrected by taking a picture of a square grid and modifying it to obtain the original rectangular geometry of the grid, similarly to the approach described by Rahman et al. [11]. The geometric correction reduces the height of the picture by 20%. This reduces the effective field of view from 98° (raw picture) to 88° (corrected picture). This correction is required before the images can be pasted (stitched) together.

#### 2.5.2. Cropping and Image Stitching

For data reduction, overlapping soil images are cropped and pasted (stitched) together to provide a single, continuous image of the surveyed domain. This process discards 30% of the top and bottom of each picture after the geometric correction and keeps 40% of the middle section (equivalent to 32% of the raw picture). Once the overlap has been removed and the geometry corrected, individual images are stitched together to create a full view image of the surveyable area. The probe step is set to 12.7 mm (0.5″) to allow this overlapping level.

### 2.6. Soil Imaging Experiments

#### 2.6.1. Example I: Detection of Non-Aqueous Phase Liquids (NAPL) Contamination in Soils

A soil column was set up to test the apparatus. A cylindrical tank was set up using a standard 6″ PVC pipe (5.5′ long), capped at the bottom using a standard PVC pipe cap (glued with PVC glue). Four ports were installed on the side of the PVC column using threaded brass connections at 12′ intervals from the bottom. The 2.5″ PVC pipe that contained the borosilicate glass portion to be surveyed was lowered into the 6″ soil column before filling up the tank with layers of different sand materials. The materials and their approximate particle diameter ranges were as follows: (i) small gravel (1.5–2.5 mm), (ii) coarse sand (1.2–1.7 mm), (iii) medium sand (0.8–1.2 mm), and (iv) fine sand (0.3–0.5 mm). The intervals at which these were used in the column were as follows: medium sand from 0 to 62.2 cm (0–24.5″) and 92.7–116.8 cm (36.5–46.0″); small gravel from 62.2 to 80 cm (24.5–31.5″); fine sand from 80 to 92.7 cm (31.5–36.5″); and coarse sand from 118.1 to 130.8 cm (46–51.5″). The tank was then filled with water to wet the sand and then drained of the water to achieve residual water saturation. A mixture of NAPLs (a mixture of actual heavy crude from a field site plus petroleum diesel and baby oil with 0.025% of a fluorescent dye) was added through the side ports of the soil column and then drained. The soil column was surveyed twice, first after adding the contaminant and later, after draining.

#### 2.6.2. Example II. Study of Plant Root Growth

A basil plant (*Ocimum basilicum*) from a local nursery was planted at a depth of approximately 10 cm (4″) on the top of the soil column described for Example I. Spanish moss (approximately 50 g) was added around the roots to aid with water retention. A portable artificial light (red and blue LED, 660 and 460 nm, respectively) was emplaced above the plant and programmed on 12 h on/off cycles. The plant was watered approximately 2 times per week until it started showing signs of growth. At that point, surveys were conducted approximately 2–3 times a week until roots started to appear on the glass portion of the dry well (time 0). The surveys continued for approximately 14 days afterwards, after significant root growth was documented.

## 3. Results

### 3.1. Example I: Detection of Non-Aqueous Phase Liquids (NAPL) Contamination in Soils

**Figure 7:**
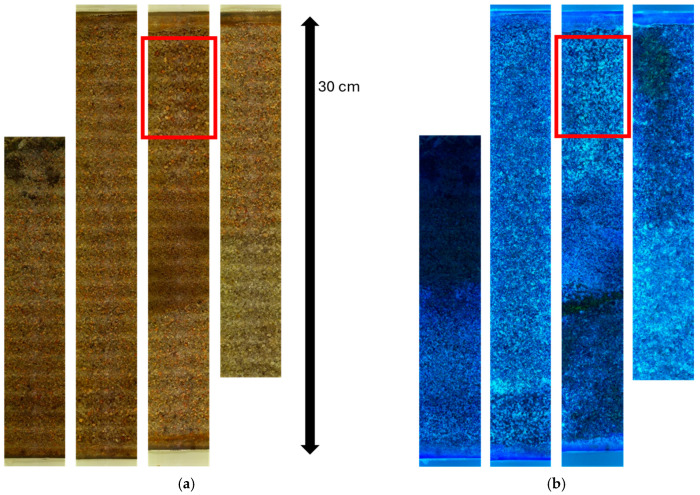
Completed survey of approximately 110 cm of soil depth (the entire surveyable length of dry well installed within the laboratory tank). (**a**) The left panel shows the stitched set of pictures obtained with the visible lights; (**b**) the right shows the pictures obtained using UV lights (365 nm), revealing the soil contaminant distribution. On each set of pictures, left to right represents ~30 cm sections of the column from top to bottom. The highlighted section is shown at a larger scale (zoomed in) in Figure 8.

The NAPL was added through the side ports of the column until surveys showed contamination in all the different types of sand used. The column was initially filled up with 12.6 L of water and then gravity drained to accommodate the added NAPL (approximate volume of 1.3 L). After the NAPL was added, water was added back to the column to simulate a contaminant under water-saturated conditions. Note that although this method might not be representative of field conditions, it was used to provide measurable NAPL contamination throughout the entire soil column. A survey was completed once the contaminant was loaded in the soil, and a second survey was performed after drainage (which was performed by gravity through the bottom port of the column). The amounts of water and non-aqueous phase liquid (NAPL) recovered were 5.2 L and 0.4 L, respectively. Figure 7 shows the survey of the entire viewable portion of the column, after having been drained from contaminant and water, under both visible lights (left) and UV (365 nm) lights.

**Figure 8:**
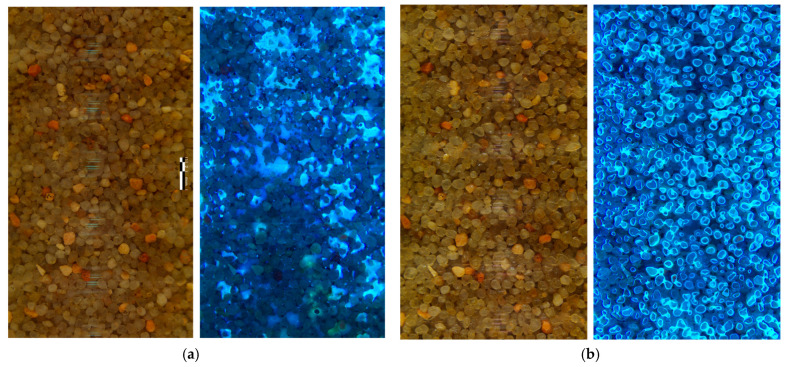
Survey detail of a 9 cm long section. (**a**) The two pictures on the left panel show the survey results under water-saturated conditions (using white and UV lights, from left to right, respectively); (**b**) the two pictures on the right panel show the survey results under water-unsaturated conditions, after draining the water and the contaminant (using white and UV lights, from left to right, respectively).

### 3.2. Example II. Study of Plant Root Growth

**Figure 9:**
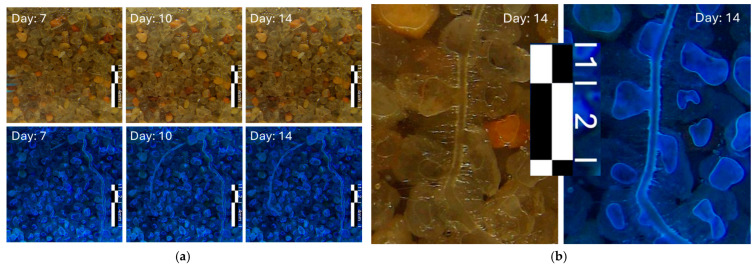
Selection of illustrative surveys of a basil plant root growth section. (**a**) The left panel shows images obtained under visible lights (top) and under UV lights (bottom) at 7, 10, and 14 days after the appearance of the first root in the image. (**b**) The right panel on the right shows a zoomed-in image with details of the root structure.

**Figure 10:**
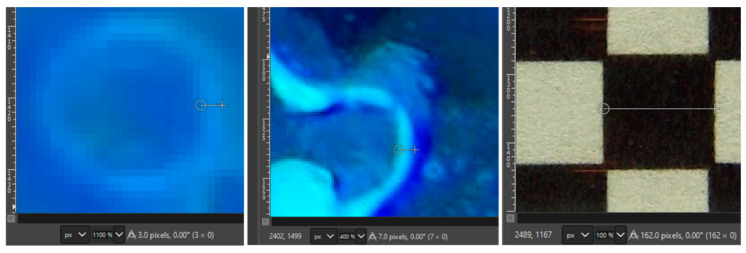
Zoomed-in picture of two LNAPL thin films to show their thickness.

The same tank used for the first experiment was used to monitor the root plant growth example. It took approximately 16 days until the basil plant root growth reached the surveyable portion of the dry well. That was considered day 0 for this report. Figure 9 shows the growth of a branch centered in the images. The root experienced approximately 2 cm of growth from 7 to 14 days. Figure 10 shows a close-up picture of the roots. A similar procedure to the one used in Example I shows that the main root branch has a diameter of 500 µm and that the finer secondary roots have a thickness of approximately 20–30 µm.

## 4. Discussion

### 4.1. Example I. Detection of Non-Aqueous Phase Liquids (NAPL) Contamination in Soils

While the close-up pictures obtained using the visible lights (revealing the soil structure) are very similar, the pictures obtained using the UV lights show a very different distribution of the contaminant. The texture of the contaminant distribution under water-saturated conditions represents a condition under which the contaminant fills full pores. Once the NAPL contaminant is drained, the contaminant no longer fills full pores but instead coats the water-coated soil particles in a continuous phase. This has been documented as caused by a change in the NAPL contaminant from non-soil wetting under water-saturated conditions to becoming a wetting fluid under water-saturated conditions [10]. This is also known as the formation of a NAPL sheen (a thin film) under water-unsaturated conditions that are more mobile than when the NAPL is surrounded by water [18]. Under drainage conditions, the remaining contaminant mass is known as residual NAPL saturation.

Note that the images obtained using the white light source showed small band reflections (glare) due to the position of the LEDs with respect to the camera (see Figure 8). These reflections are in the order of 1 mm wide and approximately 0.1 mm thick, occurring approximately every 0.3 mm near the center of the image. Because this glare is localized at the center of the images and because of its relatively small size compared to the particle size of the soil, it did not interfere with the image interpretation. Some postprocessing techniques might help remove glare, if needed (for example, see reference [11]).

The stitched images do not show significant vertical discontinuities—this supports the notion that the Position Control served the purpose of having consistently overlapping images. If improvements were needed, this Position Control system could be further improved with options like vertical sensors for improved position (for example, using an accelerometer) and/or a gyroscope (to adjust the images for the small differences in horizontal alignment, as shown in Figure 8).

The formation of thin films after draining was used to estimate the maximum resolution of the camera (see Figure 10). A checkerboard grid of 2 mm × 2 mm squares was printed on a laser printer and taped on a glass tube identical to that installed in the tank. The square dimensions were measured using the measure tool in GIMP [19]. The average 2 mm square dimensions (from 20 repetitions) were equivalent to 160.6 pixels (with a coefficient of variation of 4%). This results in an average pixel width of 12.5 µm. As a reference, the soils with the smallest particle sizes are silt and clays, with particles in the order of 20 and <5 µm, respectively [20]. Then, a similar analysis of two areas of the UV pictures with NAPL thin films yielded a sheen thickness of 3–4 pixels, equivalent to ~35–50 µm. This thickness is near or slightly below that of a human hair [21].

### 4.2. Example II. Study of Plant Root Growth

**Figure 11:**
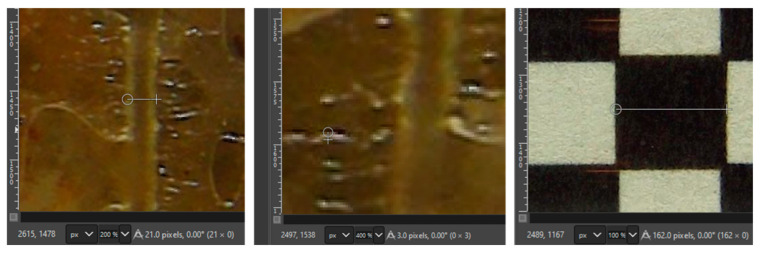
Zoomed-in picture of the plant root used to calculate thickness.

The root growth experiment showed a clear differentiation between a main plant root structure (primary) and thinner filaments (secondary root structure). As illustrated in Figure 11, a similar procedure to that used in Example I shows that the main root branch has a diameter of 500 µm and that the finer secondary roots have a thickness of approximately 20–30 µm.

### 4.3. Limitations

The main goal of this manuscript is to describe the instrument, its operation, and the postprocessing procedures. The two examples provided are offered as broad examples of potential applications, rather than rigorous analyses of soil processes. Thus, the analysis of these examples is general, rather than exhaustive. The instrument continues to be improved. This section outlines the limitations of the approach proposed; if applicable, a note about current work to address this limitation will be included in the following descriptions:

(a) The proposed imaging method is limited to the interface of the glass used for the field installation (Figure 2) and the soil. This assumes that the processes monitored using this method are representative of those occurring deeper in the soil. This might be a reasonable assumption for the examples shown here, but the assumption should be verified on a case-by-case basis for each application.
(b) Multispectral Data: In its current state, the instrument includes two sets of lights. A light source redesign is under way to accommodate additional LEDs of different excitation wavelengths to collect more multispectral data in the same survey.
(c) The images can be used to measure NAPL contaminant saturations [15] (as it is performed in other imaging methods, such as LIF [7]). This approach should be validated using a mass balance for Example I.
(d) The response spectra might be of interest, as it might reveal underlying processes [22]. For example, the fluorescent response of NAPL contaminants depends on the contaminant composition [23,24,25]. Similarly, the optical properties of plant roots might be sensitive to specific plant stressors [26]. These potential benefits might be multiplied by collecting multispectral data (see (a) above) and quantifying the optical response.
(e) The current instrument is designed to be supervised and triggered by a human handler. However, it lends itself to automation, using either preprogrammed schedules or triggered by external events.

## 5. Conclusions

This work demonstrates that a combination of a fixed port in the ground that includes a transparent section and an instrument based on a miniature camera and light sources can measure processes in the soil that change with time. The resolution of this instrument is down to about 15–20 µm (smaller than the thickness of a human hair), and the instrument can be deployed to depths down to ~15 m or more. The surveyable intervals are ~1.5 m long, and multiple intervals can be stacked within the same location. After the initial field installation, the permanent port can then be surveyed on demand with a portable instrument in a non-destructive way. These non-destructive surveys can easily be planned and programmed according to the time scale of the processes being measured. For example, root growth might occur over a few days, while NAPL distributions might follow annual river stages in some cases or tidal daily cycles in others. We hope that this instrument will aid researchers in the study of these processes.

## 6. Patents

The technology used in this work is patented (Patent US20200300761A1, Methods, Systems, and Devices for Measuring in Situ Saturations of Petroleum and NAPL in Soils).

## Figures and Tables

**Table 1:** Operating parameters for each camera and light source combinations.

	UV (365 nm)	White
Camera Operating Parameters	ISO: 200	ISO: 100
	Exposure: 60 ms	Exposure: 10 ms
LED Operating Parameters	4 LEDs × 500 mA	2 LEDs × 350 mA
	Voltage: 3.7 V	Voltage: 3.05 V
	PWM (Duty Cycle): 100%	PWM (Duty Cycle): 80%

**Table 2:** Summary of soil imaging instrument features.

Instrument Attribute	Value
Image Resolution (DPI)	1760
Camera Sensor	Sony IMX477
Camera	RGB CMOS (12 MP)
Illumination Control	Independently adjustable
Number of Light Sources	2 (white 4000 K, UV light 365 nm)
Control Mode	Programmable, automatic
Usable Tube Inner Diameter	52 mm
Usable Tube Length	Multiple intervals of 1.4 m each
Operation	Needs human interaction
Depth	~15 m maximum
Usable Radius (FOV)	88° (after geometric correction)
Focal Distance	17 mm
Lens	Wide angle (140°)
Surveying Velocity	3.2 m/h (10.52 ft/h)
Main Power Source (Battery)	12 V (portable 240 Wh)
Minimum Current Required	2.4 A
Power Consumption	53 Wh

## Data Availability

The final post-processed surveys for both experiments described are available (as submitted during manuscript submission).

## Abbreviations

The following abbreviations are used in this manuscript:

BGS	Below Ground Surface
DPI	Dots per Inch
FOV	Field of View
GUI	Graphical User Interface
LIF	Laser-Induced Fluorescence
MP	Mega Pixel
NAPL	Non-Aqueous Phase Liquids
PWM	Pulse Width Modulation
UV	Ultraviolet
